# The Value and Distribution of High-Density Lipoprotein Subclass in Patients with Acute Coronary Syndrome

**DOI:** 10.1371/journal.pone.0085114

**Published:** 2014-01-23

**Authors:** Li Tian, Chuanwei Li, Yinghui Liu, Yucheng Chen, Mingde Fu

**Affiliations:** 1 Laboratory of Endocrinology and Metabolism, West China Hospital, Sichuan University, Chengdu, Sichuan, People's Republic of China; 2 Cardiovascular Department, West China Hospital, Sichuan University, Chengdu, Sichuan, People's Republic of China; University of Leicester, United Kingdom

## Abstract

**Background:**

High-density lipoprotein (HDL) enhances cholesterol efflux from the arterial wall and exhibits potent anti-inflammatory and anti-atherosclerosis (AS) properties. Whether raised HDL levels will clinically benefit patients with acute coronary syndrome (ACS) and the value at which these effects will be apparent, however, is debatable. This study examined the HDL subclass distribution profile in patients with ACS.

**Methods:**

Plasma HDL subclasses were measured in 158 patients with established ACS and quantified by two-dimensional gel electrophoresis and immunoblotting. ACS diagnosis was based on symptoms of cardiac ischemia, electrocardiogram (ECG) abnormalities, speciality cardiac enzyme change along with presence of coronary heart disease (CHD) on coronary angiography.

**Results:**

The small-sized preβ_1_-HDL, HDL_3b_, and HDL_3a_ levels were significantly higher, and the large-sized HDL_2a_ and HDL_2b_ levels were significantly lower in patients with ACS than in those with stable angina pectoris (SAP) and in normal control subjects. Meanwhile, with an elevation in the low-density lipoprotein cholesterol (LDL-C), fasting plasma glucose (FPG), body mass index (BMI), and blood pressure (BP), and the reduction in the high density lipoprotein cholesterol (HDL-C) levels, the HDL_2b_ contents significantly decreased and the preβ_1_-HDL contents significantly increased in patients with ACS. The correlation analysis revealed that the apolipoprotein (apo)A-I levels were positively and significantly with all HDL subclasses contents; plasma total cholesterol (TC) and fasting plasma glucose (FPG) levels were inversely associated with HDL_2a_, and HDL_2b_. Moreover, the FPG levels were positively related to HDL_3c_, HDL_3b_, and HDL_3a_ in ACS patients.

**Conclusion:**

The HDL subclass distribution profile remodeling was noted in the patients with ACS. Plasma lipoprotein and FPG levels, BP, and BMI play an important role in the HDL subclass metabolism disorder for patients with ACS. The HDL subclass distribution phenotype might be useful as a novel biomarker to assist in the risk stratification of patients with ACS.

## Introduction

There is consistent epidemiological and clinical evidence showing that low high-density lipoprotein cholesterol (HDL-C) to be a strong independent risk factor for coronary heart disease (CHD) [1]. The literature also reported that CHD progression can be attenuated by elevating HDL-C levels [2], [3]. In addition, low levels of HDL-C might responsible for a significant percentage of residual cardiovascular risk in patients with CHD [4]–[6]. Acute coronary syndromes (ACS) patients in Middle East had a high prevalence of low HDL-C levels [7].

The strong inverse relationship between plasma HDL-C and atherosclerotic cardiovascular disease (CVD) provides the epidemiological bases that HDL is atheroprotective [8]. HDL promotes cholesterol efflux from the arterial wall [9] and exhibits potent anti-inflammatory and anti-atherosclerosis (AS) properties [10], whether and what degree raising HDL levels provides clinical benefit in patients with ACS, however, is currently still matter of debate [8].

It is well known that the HDL do not represent a sum of identical particles but rather are comprised from various subclasses that differ with respect to their size, density, chemical composition and other physicochemical properties. Using two-dimensional gel electrophoresis coupled with immunoblotting, HDL can be divided into large, cholesterol-rich (HDL_2a_ and HDL_2b_), small-sized (HDL_3c_, HDL_3b_, HDL_3a_, and preβ_1_-HDL) and preβ_2_-HDL [10], [11]. Epidemiological studies have shown that individual HDL subclasses are not equally atheroprotective [12], an increase content of the small-sized preβ_1_-HDL particles and a decrease content of the large-sized HDL_2b_ particles were positively and significantly associated with the risk of CHD [13], [14]. Accumulation of small preβ_1_-HDL may be a result of inefficient conversion of preβ_1_-HDL into preβ_2_-HDL or the esterification of cholesterol. Thus, higher levels of preβ_1_-HDL would have a negative impact on the anti-atherogenic potential of HDL. Large cholesterol-rich HDL_2b_ particles may be important in determining the direction of the flow of cholesterol ester (CE). In the absence of HDL_2b_ particles, HDL-CE is transferred to very low density lipoprotein (VLDL) and low density lipoprotein (LDL) by the action of cholesteryl ester transfer protein (CETP), resulting in an increase of CE in potentially atherogenic particles.

In ACS, vascular inflammation is a main factor affecting plaque vulnerability and prothrombotic state [15]. Under such inflammatory conditions, the protein and phospholipids (PL) moieties of HDL are substantially altered, therefore modifying the functional characteristics of the HDL particles [16]. HDL isolated from coronary artery disease (CAD) subjects has been shown to exert proinflammatory properties *in vitro* relative to HDL particles isolated from control subjects [17]. Therefore, it is suggested that by triggering an inflammatory response, ACS may transform HDL from antiinflammatory into a proinflammatory molecule.

To test the hypothesis that HDL particles are remodeled in ACS, we compared the distribution of HDL subclasses particles isolated from ACS patients to those isolated from stable CAD patients and normal control subjects. It has the potential to identify HDL subclass distribution profile as novel biomarkers that correlate with clinical manifestations in ACS patients.

## Methods and Subjects

### Study Design

The study was planned to investigate the HDL subclasses distribution phenotype in acute coronary syndrome (ACS) patients and also analysis and compare the phenotype in ACS patients, stable coronary heart disease (CHD) patients (stable angina pectoris, SAP) patients along with normal control subjects.

Demographic and other baseline clinical and biochemical characteristics of the patients were evaluated. Protocols were reviewed and approved by the West China Hospital ethics committee; and patients provided written informed consent prior to any study-related procedure.

### Subjects

235 patients with established CHD participated in this study. These patients were hospitalized in the Cardiovascular Department of the West China Hospital, Sichuan University. Diagnosis was based on clinical history and was confirmed by quantitative coronary angiography (QCA). The results of angiographic examination were regarded positive for coronary atherosclerosis only if one or more major coronary arteries(right coronary, left main coronary, left anterior descending, and circumflex) had at least 50% stenosis of the luminal area. The angiogram was reviewed by two physicians.

Exclusion criteria included: uncontrolled diabetes mellitus, diabetes mellitus requiring insulin therapy, secondary causes of hyperlipidemia, such as uncontrolled primary hypothyroidism with thyroid stimulating hormone (TSH) greater than 5.5 uIU/ml; impaired renal function (creatinine >2.0 mg/dL) or nephritic syndrome; the presence of active liver disease or hepatic dysfunction; clinically significant hematology abnormalities. Subjects current or recent history consumption of more than 14 alcoholic drinks per week along with use of immunosuppressive agents also excluded in the present study. The patients were concurrently taking antihypertensive medication (80% patients using calcium channel blockers; 85% patients using beta blockers; 75% patients using diuretics); 70% patients were also using other antihypertensive therapies, and the medications did not change over the course of the study.

The 158 patients in ACS subgroups including UA (ustable angina) and AMI (acute myocardial infarction) according to the American College of Cardiology (ACC)/American Heart Association (AHA) Guidelines. The diagnosis of ACS was confirmed by clinical evaluation by the cardiologist, including the signs, symptoms of cardiac ischemia, electrocardiogram (ECG) abnormalities (ST-segment elevation myocardial infarction, STEMI and non-ST-segment elevation myocardial infarction, NSTEMI) speciality cardiac enzyme (CKMB), cardiac troponic (cTn) change along with presence of CHD on coronary angiography. As comparison groups, we also examined the 77 patients with stable CHD that is stable angina pectoris(SAP) subgroups and 86 subjects normal healthy control (Normal controls) subgroups. Normal controls subgroups consisted of age-matched normal healthy subjects from West China Center of Medical Science, Sichuan University. These subjects had no CHD as recorded by coronary angiography. The waist, hip circumferences were not measured for the normal controls subjects, and some subjects blood pressure (BP) examination recorded missed.

The effect of LDL-C and HDL-C levels on the HDL subclass distribution was assessed in ACS patients.

The reference levels of the plasma lipids were defined by following guidelines from the Adult Treatment Panel III (ATP-III) of the National Cholesterol Education Program (NCEP), that is: desirable LDL-C (<130 mg/dL; 3.34 mmol/L), borderline LDL-C (130–160 mg/dL; 3.36–4.11 mmol/L), high LDL-C (≥160 mg/dL; 4.14 mmol/L), low HDL-C (<40 mg/dL; 1.03 mmol/L), middle HDL-C (40–60 mg/dL; 1.03–1.55 mmol/L), and high HDL-C (≥60 mg/dL; 1.55 mmol/L).

Body mass index (BMI) was calculated as the ratio of weight (kg) to the square of height (m^2^). Finally, according to the recommended criterion for BMI of the Working Group on Obesity in China (WGOC) under the support of International Life Sciences focal point in China, BMI<18.5 kg/m^2^ were defined as underweight, BMI 18.5–24 kg/m^2^ were defined as desirable weight, 24<BMI≤28 kg/m^2^ were defined as overweight.

### Angiographic Assessment of Coronary Arteries

Quantitative coronary angiography (QCA) was performed at the cardiology department of our hospital. QCA was carried out by resident physicians using the Judkins method, specifically through arteria radialis or arteria cruralis, multiposition projection. QCA was performed at baseline according to standard methods. A minimum of 3 sets of orthogonal views of the left coronary artery and 1 of the right coronary artery were obtained from each subject. Analysis of angiograms was performed with a previously validated system of cine-projection. Briefly, the reference, minimal diameter (the point of greatest narrowing), and the average luminal diameters were obtained for 10 proximal epicardial coronary artery segments. The mean minimal coronary artery diameter was calculated in each subject as the average of the minimal luminal diameter in the 10 coronary segments.

### Specimens

Fasting (12 h) blood samples were collected in tubes containing 0.1% EDTA and centrifuged at 3,000 r for 20 min at 4°C to obtain plasma. Plasma samples were stored at 4°C and used within 24 h for lipid and apolipoprotein analyses. Aliquots of each plasma sample were stored at −70°C for the determination of HDL subclasses.

### Plasma Lipid and Apolipoprotein Analyses

Plasma concentrations of TG, TC, HDL-C, and LDL-C, along with fasting plasma glucose (FPG), apoA-I, and apoB-100 values were measured for all of the subjects using automated standardized equipment by the Clinical Laboratory of West China Hospital, Sichuan University.

### HDL Subclasses Analyses

The subclass distribution of HDL was determined with 2-dimensional (2-D) gel electrophoresis and subsequent immunodetection as described previously [10]. In brief, 10 ul of plasma was applied to 0.7% agarose gel and electrophoresis in the first dimension. After electrophoretic separation of lipoproteins, further separation by electrophoresis was carried out along the 2–30% nondenaturing polyacrylamide gradient gel in the second dimension. To determine HDL subclasses, Western blotting was conducted after 2-D gel electrophoretic, plasma proteins and molecular markers were electrophoretically transferred to polyvinylidene (PVDF) membranes, stained with 0.1% Ponceau S, and the position of molecular standard protein bands was labeled with a pencil. They were then destained by diffusion and 5%bovine serum albumin (BSA) was used to recover the membrane, followed by a reaction with horseradish peroxidase (HRP)-labeled goat antihuman apoA-I immunoglobulin G (IgG). The relative concentration of each HDL subclass was calculated as the percentage of total plasma apoA-I according to the density of each spot. HDL particle sizes were calibrated using a standard curve that included bovine serum albumin, ferritin, and thyroglobulin (Pharmacia Uppsala, Sweden).The relative percent concentration of each HDL subclass was multiplied by the apoA-I concentration in the sample to yield the relative concentrations of each HDL subclass of apoA-I (mg/L). The intra-assay variation (N = 5) of the specific HDL subclasses was 9.4% (preβ_1_-HDL), 9.8% (preβ_2_-HDL), 4.9% (HDL_3c_), 6.2% (HDL_3b_), 7.3% (HDL_3a_), 11.1% (HDL_2a_), and 7.9% (HDL_2b_).

### Statistical Analysis

The Kolmogorov-Smirnov test was used to determine the normality of distributions of the variables. Non-Gaussian distribution was transformed to Gaussian distribution using the natural logarithm conversion. All values are presented as mean ± standard deviation (SD). Significant differences between the groups were analyzed by one-way analysis of variance. Pearson correlation analysis was used to estimate the correlation between plasma lipoproteins and the changes in HDL subclass profile among ACS patients. Differences were considered statistically significant at P<0. 05. All statistical analyses were performed using the statistical package SPSS (Version 17.0, SPSS Inc).

## Results

### Demographic data in ACS, SAP and Normal Control Groups

Table 1 showed that the mean age, waist, hip, systolic blood pressure (SBP) were significantly higher in ACS patients versus those in SAP patients. In addition, the levels of FPG; and waist were significantly increased for women patients than those for men patients in ACS group; meanwhile, the levels of FPG; and hip were significantly decreased for women patients versus those of men patients in SAP group.

**Table 1:** Demographic Data in ACS, SAP, and Normal Control Groups.

	ACS Group			SAP Group			Normal Control Group		
	Total (n = 158)	Men (n = 87)	Women (n = 71)	Total (n = 77)	Men (n = 45)	Women (n = 32)	Total (n = 86)	Men (n = 53)	Women (n = 33)
**Age(yr)**	65.9±8.8	65.1±8.8	67.0±9.0	63.1±8.5^b^ *	62.2±7.2	64.1±8.1	62.1±8.7^b^ †	62.1±9.5	60.9±8.3
**BMI(kg/m^2^)**	25.6±2.7	25.1±2.5	26.0±2.7	24.3±2.5	24.5±2.4	24.0±2.3	22.5±2.0^b^ *	22.9±2.2	22.3±2.1
**Waist(cm)**	93.4±9.0	91.3±7.8	95.6±8.3^a^ *	89.3±8.8^b^ *	90.1±7.8	88.3±7.5	N	N	N
**Hip(cm)**	94.4±7.5	94.0±7.6	95.1±7.8	90.1±7.1^b^ *	92.3±6.9	88.1±6.6^a^ *	N	N	N
**SBP(mmHg)**	139.1±18.7	134.9±17.2	143.3±19.2	123.1±17.1^b^ *	122.9±13.9	124.3±15.6	N	N	N
**DBP(mmHg)**	77.1±9.8	75.1±9.0	79.7±9.6	72.2±9.3	70.1±9.0	74.5±9.8	N	N	N
**FPG(mmol/L)**	6.6±2.0	6.1±1.8	7.1±2.3^a^ *	6.1±1.7	6.5±2.0	5.7±1.9^a^ *	5.0±1.8^b^ † c *	5.0±1.9	4.9±1.7

ACS, acute coronary syndromes; SAP, stable angina pectoris; BMI, body mass index; SBP, systolic blood pressure; DBP, diastolic blood pressure; FPG, fasting plasma glucose.

Data are presented as Mean ± S.D.

* *P*<0.05,

† *P*<0.01,

§ *P*<0.01.

a Compared with the corresponding men in the same ACS and SAP groups, respectively.

b Compared with the corresponding total patients in ACS group.

c Compared with the corresponding total patients in SAP group.

Due to the waist, hip, and blood pressure (BP) data were missed for subjects in normal control group, the current results showed that the mean age, and body mass index (BMI) were significant lower for subjects in normal control group than those for patients in ACS group. The levels of FPG were significant lower for subjects in normal control group compared with those for patients both in ACS and SAP groups.

### Plasma lipids, lipoproteins, apolipoproteins concentrations and HDL subclasses contents in ACS, SAP and Normal Control Groups

Table 2 presented that the values for plasma lipids, lipoproteins, apolipoproteins, and HDL subclasses contents of patients between in these two groups. For patients in ACS group, the concentrations of plasma TG, TC, LDL-C, apoB100; and the ratios of TG to HDL-C, TC to HDL-C, and apoB100 to apoA-I were significantly higher; in contrast, concentrations of apoA-I and HDL-C were significantly lower when compared with those for patients in SAP group.

**Table 2:** Concentrations of Plasma Lipids, Lipoproteins, Apolipoproteins and HDL Subclasses Contents in ACS, SAP, and Normal control Groups.

	ACS Group			SAP Group			Normal Control Group		
	Total (n = 158)	Men (n = 87)	Women (n = 71)	Total (n = 77)	Men (n = 45)	Women (n = 32)	Total(n = 86)	Men (n = 53)	Women (n = 33)
TG(mmol/L)	2.2±0.8	2.0±0.6	2.5±0.8^a^ *	1.7±0.6^b^ *	1.9±0.6	1.7±0.5	1.2±0.2^b^ § c *	1.3±0.2	1.1±0.2
TC(mmol/L)	4.4±1.0	4.1±1.0	4.6±1.1^a^ *	3.8±1.0^b^ *	4.0±1.0	3.7±0.9	5.0±0.9^b^ * c §	5.0±1.0	4.8±0.9
LDL-C(mmol/L)	3.4±1.0	3.3±1.0	3.6±1.0	2.8±0.7^b^ *	3.1±0.9	2.4±0.7^a^ *	3.0±0.7^b^ *	3.1±0.9	2.8±1.0
HDL-C(mmol/L)	1.1±0.2	1.1±0.2	1.1±0.2	1.3±0.2^b^ *	1.2±0.2	1.4±0.2^a^ *	1.4±0.4^b^ †	1.3±0.3	1.6±0.2^a^ †
TG/HDL-C	2.1±0.2	1.7±0.3	2.5±0.5^a^ †	1.3±0.3^b^ †	1.5±0.3	1.1±0.2^a^ *	0.8±0.3^b^ § c †	1.0±0.4	0.7±0.3^a^ *
TC/HDL-C	3.8±0.5	3.3±0.7	4.4±1.0^a^ †	2.9±0.4^b^ †	3.3±0.6	2.6±0.4^a^ †	3.3±0.7^b^ * c *	3.7±0.6	2.9±0.4^a^ †
LDL-C/HDL-C	3.3±0.4	3.2±0.4	3.5±0.5	2.1±0.3^b^ §	2.6±0.3	1.7±0.2^a^ †	2.0±0.4^b^ §	2.2±0.4	1.7±0.3^a^ *
ApoA-I(g/L)	1.1±0.2	1.2±0.2	1.0±0.2^a^ †	1.3±0.3^b^ †	1.2±0.2	1.4±0.2^a^ †	1.3±0.2^b^ †	1.2±0.2	1.3±0.3
ApoB-100(g/L)	0.7±0.2	0.6±0.2	0.8±0.2^a^ *	0.6±0.2^b^ *	0.7±0.2	0.5±0.2^a^ *	0.6±0.2^b^ *	0.7±0.2	0.6±0.2
ApoB-100/A-I	0.6±0.2	0.5±0.2	0.7±0.2^a^ †	0.5±0.2^b^ *	0.6±0.2	0.4±0.2^a^ †	0.5±0.2^b^ *	0.5±0.2	0.4±0.2
Preβ_1_-HDL(mg/L)	108.8±12.6	96.4±10.3	121.3±21.1^a^ *	91.3±10.3^b^ *	93.3±10.1	89.6±9.3	78.9±16.3^b^ †	84.3±17.7	74.6±14.3
Preβ_2_-HDL(mg/L)	54.4±6.0	53.4±5.6	55.4±5.3	51.5±5.7	52.6±5.1	50.5±5.0	56.8±7.0	56.2±6.2	58.2±6.6
HDL_3c_(mg/L)	90.4±11.4	87.6±10.4	93.3±13.7	84.6±9.4	88.8±10.2	81.1±9.7	70.8±7.1^b^ *	71.4±9.4	68.3±7.6
HDL_3b_(mg/L)	155.2±27.1	142.9±29.4	167.5±30.3^a^ *	135.3±21.3^b^ *	144.6±31.8	128.2±21.5^a^ *	133.7±23.8^b^ *	143.0±21.9	120.4±25.4^a^ *
HDL_3a_(mg/L)	286.9±40.6	271.3±43.3	302.3±45.7^a^ †	247.9±31.3^b^ †	256.8±46.7	240.7±37.9^a^ *	242.5±23.8^b^ †	255.9±36.1	238.1±30.5^a^ *
HDL_2a_(mg/L)	215.7±30.9	231.1±31.6	200.4±27.3^a^ †	275.7±38.1^b^ §	263.7±45.3	287.3±51.9^a^ *	280.4±48.1^b^ §	270..9±48.3	289.1±47.3^a^ *
HDL_2b_(mg/L)	260.5±60.6	290.5±47.3	235.4±31.6^a^ †	339.1±66.6^b^ §	311.9±61.6	357.1±67.7^a^ †	388.5±63.6^b^ § c †	376.4±58.3	402.3±64.4^a^ *

ACS, acute coronary syndromes; SAP, stable angina pectoris; TG, triglyceride; TC, total cholesterol; LDL-C, low density lipoprotein cholesterol; HDL-C, high density lipoprotein cholesterol; ApoA-I, apolipoproteinA-I; ApoB-100, apolipoproteinB-100;

Data are presented as Mean ± S.D.

* *P*<0.05,

† *P*<0.01,

§ *P*<0.001.

a Compared with the corresponding men in the same ACS and SAP groups, respectively.

b Compared with the corresponding total patients in ACS group.

c Compared with the corresponding total patients in SAP group.

For the distribution of HDL subclasses, small-sized preβ_1_-HDL, HDL_3b_, and HDL_3a_ were obviously higher (*P*<.05. *P*<.05 and *P*<.01, respectively); however, large-sized HDL_2a_ and HDL_2b_ were significantly lower (*P*<.001 and *P*<.001, respectively) in ACS patients versus those in SAP patients.

Women patients had atherogenic lipoproteins profile (TG, TC increased, HDL-C decreased) and higher small-sized preβ_1_-HDL, HDL_3b_, and HDL_3a_ (*P*<.05, *P*<.05 and *P*<.01, respectively), but lower large-sized HDL_2a_ and HDL_2b_ (*P*<.01 and *P*<.01, respectively) than those for men patients in ACS group. Contrary to the plasma lipoproteins and HDL subclasses distribution profile change between in women and men patients with ACS group; the plasma lipoproteins and HDL subclasses distribution display opposite changes for women patients in comparison with those for men patients in SAP group.

In comparison with the subjects in normal control group, the concentrations of TG, TC, LDL-C (only in ACS), apoB-100(only in ACS), and the ratios of TG/HDL-C, TC/HDL-C, LDL-C/HDL-C(only in ACS), and apoB-100/A-I (only in ACS) were increased markedly, however, those of HDL-C, and apoA-I (only in ACS) were reduced markedly for patients both in ACS and SAP groups. The phenotype of HDL subclasses for the patients in ACS and SAP groups along with the subjects in normal control group presented that the patients in ACS group had higher contents of preβ_1_-HDL (*P*<.01), HDL_3_ (HDL_3c_, HDL_3b_, HDL_3a_) (*P*<.05, *P*<.05, and *P*<.01, separately) and lower those of HDL_2_ (HDL_2a_, HDL_2b_) (*P*<.01, and *P*<.01, separately) than the subjects in normal control group. Comparison the HDL subclasses distribution between in SAP patients and Normal control subjects, there is a decreasing obvious in HDL_2b_ (*P*<.01) for patients in SAP group and other HDL subclasses contents did not the difference significant observed between in these two groups.

### Correlation coefficients between HDL subclasses and plasma lipids, apolipoproteins, other parameters in ACS patients

Correlation between HDL subclasses and lipids, apos along with other parameters in ACS patients showed that plasma TC levels were negatively correlated with large-sized HDL_2a_, HDL_2b_. Of note, plasma TG levels were positively correlated with HDL_3c_; the levels of FPG were positively associated with HDL_3c_, HDL_3b_, and HDL_3a_; but inversely with HDL_2a_ and HDL_2b_. Moreover, apoA-I were positively correlated with all HDL subclasses (Table 3).

**Table 3:** Assessment of Relationship between Plasma Lipid, Lipoprotein, Apolipoprotein and HDL Subclasses Contents in ACS Patients.

ACS	TG	TC	HDL-C	LDL-C	ApoA-I	ApoB-100	FPG
Preβ_1_-HDL	.146	.108	.106	.061	.338**	.106	.084
Preβ_2_-HDL	.057	.077	.066	.070	.248*	.094	.028
HDL_3c_	.190*	.149	.100	.108	.432**	.114	.187*
HDL_3b_	.098	.184	.132	.175	.457**	.123	.141*
HDL_3a_	.108	.141	.101	.180	.470**	.158	.219**
HDL_2a_	−.129	−.202*	.153	−.138	.538**	−.179	−.303
HDL_2b_	−.145	−.240*	.169	−.197*	.541**	−.189	−.379**

ACS, acute coronary syndromes; TG, triglyceride; TC, total cholesterol; HDL-C, high density lipoprotein cholesterol; LDL-C, low density lipoprotein cholesterol; ApoA-I, apolipoproteinA-I; ApoB-100, apolipoproteinB-100; FPG, fasting plasma glucose.

* *P*<0.05,

** *P*<0.01,

### Characteristics of major HDL subclasses (Preβ_1_-HDL, and HDL_2b_) distribution in ACS patients according to plasma LDL-C and HDL-C concentrations

**Figure 1:**
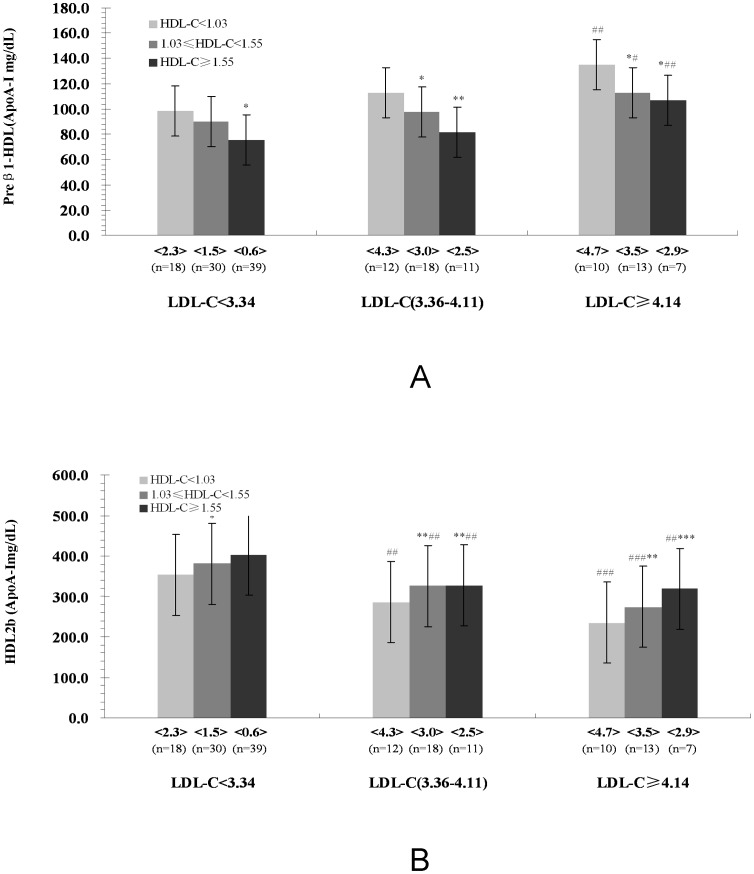
Characteristics of Major HDL Subclasses (Preβ_1_-HDL, and HDL_2b_) Distribution in ACS Patients According to Plasma LDL-C and HDL-C Concentrations. Valued are expressed as means. The values in the < > denote the LDL-C/HDL-C ratio; N: number; ^*^ *P*<0.05, ^**^ *P*<0.01, ^***^ *P*<0.001, compared with the HDL-C<1.03 mmol/L subgroup within the same LDL-C group. ^#^ *P*<0.05, ^##^ *P*<0.01, ^###^ *P*<0.001 compared with the same HDL-C subgroup within the LDL-C<3.34 mmol/L group.

As shown in Fig. 1(A), compared to the patients in the low HDL-C (<1.03 mml/L) subgroup, preβ_1_-HDL contents decreased significantly but HDL_2b_ contents increased significantly for patients in the corresponding both of middle HDL-C(1.03–1.52 mmol/L) and high HDL-C(≥1.55 mmol/L) subgroups, among in desirable LDL-C(<3.34 mmol/L), borderline LDL-C and high LDL-C groups.

Moreover, the patients in the borderline and high LDL-C groups, an elevation of preβ_1_-HDL (high LDL-C group) and a reduction of HDL_2b_ were evident in the low, middle, and high HDL-C subgroups in comparison with those for patients in corresponding HDL-C subgroup Fig. 1(B).

### Characteristics of major HDL subclasses (Preβ_1_-HDL, and HDL_2b_) distribution in ACS patients according to SBP and DBP levels

**Figure 2:**
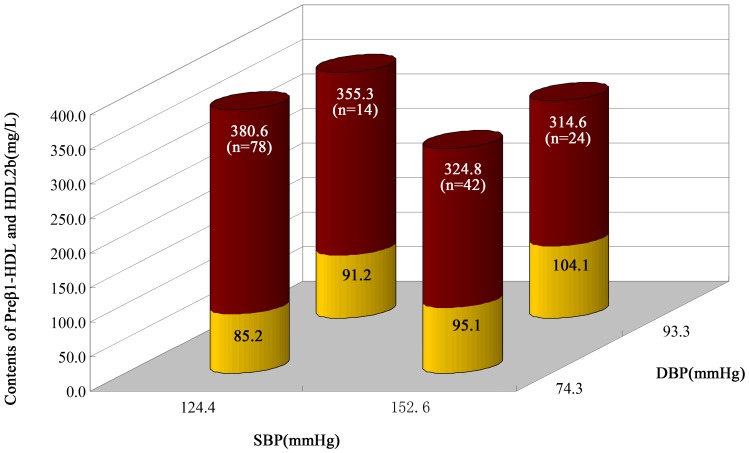
Characteristics of Major HDL Subclasses (Preβ_1_-HDL, and HDL_2b_) Distribution in ACS patients according to SBP and DBP Levels. In this chart, the red column stands for the contents of HDL_2b_ and the yellow column stands for those of preβ_1_-HDL.

Fig. 2 presented that dichotomy the levels of SBP (140 mmHg) and DBP (90 mmHg) in ACS patients, and their corresponding contents of preβ_1_-HDL and HDL_2b_. Compared with the patients in (<140/90 mmHg group), the contents of preβ_1_-HDL increased significantly, however, those of HDL_2b_ decreased significantly for patients in (≥140/90 mmHg group)(Fig. 2).

### Characteristics of major HDL subclasses (Preβ_1_-HDL, and HDL_2b_) distribution in ACS patients according to FPG and BMI

**Figure 3:**
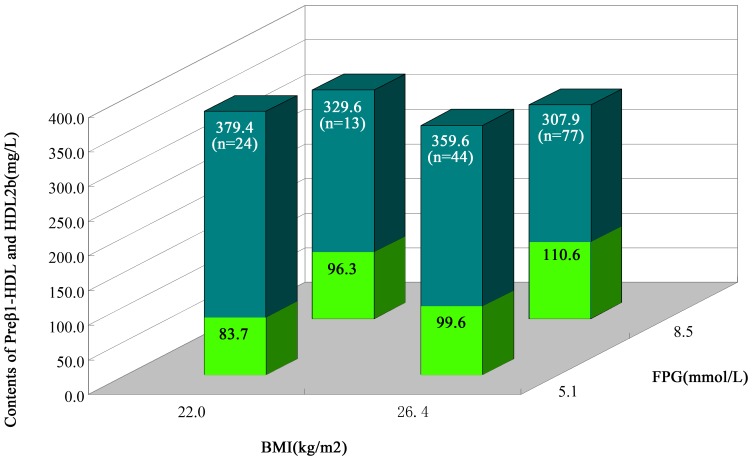
Characteristics of Major HDL Subclasses (Preβ_1_-HDL, and HDL_2b_) Distribution in ACS patients According to FPG and BMI. In this chart, the blue column stands for the contents of HDL_2b_ and the green column stands for those of preβ_1_-HDL.

The FPG levels of 6.11 mmol/L and BMI 24 kg/m^2^ were used as the cutpoints. Analyzing the relationship between the FPG levels, BMI, and the alteration of HDL subclasses showed that, in comparison with FPG<6.11 mmol/L along with BMI<24 kg/m^2^ patients, the patients in FPG≥6.11 mmol/L along with BMI≥24 kg/m^2^ had significantly higher small-sized preβ_1_-HDL, but lower large-sized HDL_2b_(Fig. 3).

## Discussion

ACS encompasses unstable angina (UAP), non-ST segment elevation myocardial infarction (NSTEMI), and ST segment elevation myocardial infarction (STEMI). Between 10% and 30% of ACS subjects experience recurrent cardiovascular (CV) events within one year, including CV death, recurrent myocardial infarction (MI), or stroke [18]. Approximately 50% of patients who present with ACS have reduced HDL-C levels [19]. Additionally, HDL-C levels at the presentation of ACS predict future risk of death or major adverse CV event [20]. In accordance with the high residual risk, new therapeutic targets and approaches are required to treat ACS patients. The HDL becomes the potential target.

Plasma lipoproteins are implicated in the genesis of atherosclerosis (AS) and lipid content of AS plaques are related to their vulnerability to rupture and trigger ACS. After ACS, the chance of recurrent complications varies according to clinical, electrocardiographic (ECC) and laboratory characteristics identified as risk predictors [21], [22]. However, the simple idea of using plasma lipoproteins as risk predictors has not been sufficiently assessed in the clinical scenario. Studies have identified independent predictors and validated risk scores did not test plasma lipids and lipoproteins, especially plasma lipoproteins subclass distribution phenotype as potential predictors. The sole evidence is provided by Olsson *et al* [20], who demonstrated that HDL-C predicted coronary events in a follow-up of 16 weeks after NSTEMI. However, the predictive value of plasma HDL subclasses distribution for ACS in-hospital was not evaluated.

We therefore tested the hypothesis that HDL particles subclasses are remodeled by comparing the HDL subclasses distribution of ACS, SAP patients, and normal controls subjects. The present study found that the concentrations of plasma TG, TC, LDL-C, and apoB100; and the ratios of TG to HDL-C, TC to HDL-C along with apoB100 to apoA-I were significantly higher; in contrast, concentrations of apoA-I and HDL-C were significantly lower for patients in ACS group compared with those for patients in SAP group and subjects in normal control group.

For the distribution of HDL subclasses, the small-sized preβ_1_-HDL, HDL_3c_ (only in normal control group), HDL_3b_, and HDL_3a_ contents were obviously higher; however, large-sized HDL_2a_ and HDL_2b_ contents were significantly lower for patients in ACS group versus those for patients in SAP group and subjects in normal control group. The profile of HDL subclass distribution remodeling in ACS patients might be involved in the increased TG, TC and LDL-C and decreased apoA-I together with HDL-C levels. Several lines of evidence suggested that enhanced CETP and hepatic lipase (HL) activities are correlated with HDL-C levels [23], [24]. CETP exchanges CE of HDL_2_ with TGs of VLDL and LDL. The HDL-derived CE is removed from the circulation via the LDL receptor pathway. TGs in HDL are hydrolyzed by HL. The concerted action of CETP and HL converts larger HDL_2_ into smaller HDL_3_ and releases lipid-free apoA-I and/or preβ_1_-HDL.

It has been reported that the concentrations of TG are inversely associated with the lipoprotein lipase (LPL) and lecithin cholesterol acyltransferase (LCAT) activities [25], [26]. LPL plays an important role in hydrolyzing TG transported in chylomicron (CM) and VLDL particles. When catabolized by LPL, CM and VLDL release TG, TC, PL, apoA-I and apoCs, subsequent binding of these products to HDL_3_ results in formation of HDL_2_ particles. ApoA-I can activate the LCAT, and LCAT may catalyze unesterified cholesterol to CE and promote the conversion of preβ_1_-HDL and HDL_3_ to HDL_2_, hence, the increased of TG levels along with the reduction of apoA-I levels resulting in the increase contents of small-sized HDL particles and decrease contents of large-sized HDL particles.

According to the results by coronary angiography and clinical manifestations, the CHD patients usually divided into ACS groups that including UAP and AMI patients and SAP groups. The SAP is a chronic, and relative stable process, but the ACS presents acute and unstable process. Compared with the normal control group, almost all HDL subclasses contents significantly change in ACS group, but, the HDL subclasses contents did not difference except for HDL_2b_ in SAP group. It is well known that the most prominent role of HDL is its role in the reverse cholesterol transport (RCT) process by which excess cholesterol is transported from peripheral cells to the liver metabolism. The cholesterol uptake from macrophages through ABC-transporters avoid the formation of foam cells in the AS lesion that is the first steps in the pathogenesis of AS. It has been postulated that RCT is the metabolic process that converts nascent preβ_1_-HDL into mature α-HDL, following the steps preβ_1_-HDL→preβ_2_-HDL→HDL_3_→HDL_2_. The distribution of HDL subclasses could be reflecting the efficiency of and speed of RCT.

Based on our findings, we proposed that HDL subclasses distribution profile modified during ACS. In this case, the current work supported the increased contents of preβ_1_-HDL and impeded the formation of cholesterol-rich mature HDL_2_ particles in ACS patients which may predispose to the development of AS.

Many studies demonstrating that various factors changes that have been shown to confer cardiovascular protection (such as plasma lipoproteins, obesity, DM (diabetic mellitus) and hypertension). Data obtained in this work displayed that among the ACS patients, increased contents of small-sized preβ_1_-HDL and decreased contents of large-sized HDL_2b_ with the elevation of LDL-C, BMI, FPG, BP along with the reduction of HDL-C levels.

In accordance with the current data which suggested that in clinical practice, it is benefit from combination therapy with lipid-lowering, glucose-lowering, and controlling for BP, and lose weight for ACS patients with abnormal HDL subclass distribution.

In one word, the current results implied that the profile of HDL subclasses distribution have remodeled in ACS patients. Plasma lipoproteins, FPG, BP, and BMI play the important role in HDL subclasses metabolism disorder for patients with ACS. The phenotype of HDL subclasses distribution might be useful as a novel biomarker which can assist in risk stratification in ACS patients.
